# Physical Activity in Patients With Heart Failure During and After COVID-19 Lockdown: Single-Center Observational Retrospective Study

**DOI:** 10.2196/30661

**Published:** 2022-04-19

**Authors:** Francesco Maria Angelo Brasca, Maria Carla Casale, Fabio Lorenzo Canevese, Giovanni Tortora, Giulia Pagano, Giovanni Luca Botto

**Affiliations:** 1 Department of Electrophysiology and Clinical Arrhythmology Azienda Socio Sanitaria Territoriale Rhodense Milano Italy

**Keywords:** heart failure, physical activity, COVID-19, remote monitoring, implantable cardiac device, monitoring, exercise, surveillance, lockdown, cardiovascular, heart, retrospective, burden

## Abstract

**Background:**

The COVID-19 pandemic forced several European governments to impose severe lockdown measures. The reduction of physical activity during the lockdown could have been deleterious.

**Objective:**

The aim of this observational, retrospective study was to investigate the effect of the lockdown strategy on the physical activity burden and subsequent reassessment in a group of patients with heart failure who were followed by means of remote monitoring.

**Methods:**

We analyzed remote monitoring transmissions during the 3-month period immediately preceding the lockdown, 69 days of lockdown, and 3-month period after the first lockdown in a cohort of patients with heart failure from a general hospital in Lombardy, Italy. We compared variation of daily physical activity measured by cardiac implantable electrical devices with clinical variables collected in a hospital database.

**Results:**

We enrolled 41 patients with heart failure that sent 176 transmissions. Physical activity decreased during the lockdown period (mean 3.4, SD 1.9 vs mean 2.9, SD 1.8 hours/day; *P*<.001) but no significant difference was found when comparing the period preceding and following the lockdown (–0.0007 hours/day; *P*=.99). We found a significant correlation between physical activity reduction during and after the lockdown (*R^2^*=0.45, *P*<.001). The only significant predictor of exercise variation in the postlockdown period was the lockdown to prelockdown physical activity ratio.

**Conclusions:**

An excessive reduction of exercise in patients with heart failure decreased the tolerance to exercise, especially in patients with more comorbidities. Remote monitoring demonstrated exercise reduction, suggesting its potential utility to encourage patients to maintain their usual physical activity levels.

## Introduction

The impact of the COVID-19 pandemic on health systems forced several European governments to impose severe lockdown rules to limit virus contagion. In Italy, since February 21, 2020 (the day of the first case identification), the dramatic spread of virus infection, particularly in the Lombardy region, induced the government to approve a national lockdown. The lockdown condition forbids people to travel and to frequent public spaces, except in cases of specific working conditions or for supplying food and essential goods.

Possible deleterious effects associated with these measures include economic slowdown, stop of schooling, and psychological distress [1]. Nevertheless, reducing the occupation of intensive care units is mandatory to limit the number of deaths due to COVID-19 [2].

In the lockdown period, the number of in-office visits was reduced due to patients’ fear of contagion and to guarantee social distancing in the ambulatory waiting rooms.

Remote control is a well-defined follow-up system that expert consensus documents have endorsed as a possible alternative to in-office device control [3]. This technology was implemented in the last few years and became an important resource, even more so during the COVID-19 pandemic when patients were recommended to stay away from hospitals as much as possible [4,5].

Substantial information is available from device remote transmissions, including the quantification of patients’ physical activity (PA). As the patient moves, a sensor detects body motion and generates a signal proportional to the amplitude and frequency of movement [6].

Regular training remains a low-appreciated therapy in patients with heart failure (HF) due to medical concern about safety for such patients and poor medical evidence of strong benefits [7]. Moreover, the patient can also be worried about engaging in PA owing to comorbidities, being elderly, and logistic considerations.

Nevertheless, the possible benefits of exercise include the preservation of sympathetic nerve and arterial baroreflex control, and improvement of the transport and utilization of oxygen in the skeletal muscle [8]. These physiological effects were associated with lower mortality and hospitalization in the HF-ACTION trial [9], although a subsequent meta-analysis failed to show a mortality benefit [10].

For these reasons, the reduction of PA during the lockdown could have been deleterious, in particular for patients with HF. It has been shown that a large portion of patients (49%) could fail to recover their usual PA after the lockdown period [11], and the frailty status of older patients could worsen because of stopping their cardiac rehabilitation [12].

The aim of this study was to investigate the effect of the lockdown strategy on the PA burden and subsequent reassessment in a group of patients with HF who were followed by means of remote monitoring.

## Methods

### Study Design

This was a retrospective, single-center, observational investigation on the impact of the lockdown strategy on average daily PA in patients with HF. We enrolled all 454 patients who were implanted with a device (cardiac resynchronization therapy [CRT] or implantable cardioverter defibrillator [ICD]) and were followed by means of remote monitoring in our center, located in the Milan area, Lombardy region, Italy.

We excluded 190 patients who had no history of HF. We only included devices implemented with the Cardiac Compass (Medtronic Inc) software, due to differences in the diagnostic tools for estimating PA by devices from other manufacturers. Thus, we analyzed the data from the remaining 112 patients.

Patients whose remote monitoring period started after December 8, 2019, were excluded (n=44), as well as those who stopped remote monitoring within 3 months after the end of lockdown for a nonclinical reason (n=21). We also excluded patients with very low PA in the 3 months before lockdown, arbitrarily defined as less than 0.5 hours/day, because of the supposed negligible effect of the limitation introduced by government measures (n=6).

We analyzed the average daily PA in patients with HF during a period that included the 3 months immediately preceding the onset of the lockdown (from December 8, 2019, to March 8, 2020), the 69 days of lockdown (March 8 to May 18, 2020), the 3 months after the end of lockdown (May 19 to August 18, 2020), and the same period in 2019 (May 19 to August 18, 2019).

### Ethical Considerations

This is a retrospective analysis of data collected by previously implanted device. Before device implantation, every patient had a meeting with a medical doctor and signed a specific consent for device implantation and anonymized data collection for research purposes.

### Data Collection

We collected demographics for all patients. Echocardiographic data were obtained from the hospital database. Clinical data such as hypertension, diabetes, atrial fibrillation (AF), and ischemic cardiac disease were collected from our database of hospital discharges, in-office visits, and emergency department accesses. The last blood examination available was considered if more recent than 6 months.

### Measures

According to our follow-up protocol for remote monitoring, patients had scheduled periodical device remote transmissions (at least every 3 months) and the devices could send alarm transmissions due to prespecified alarm conditions. Alarms were delivered for any electrical malfunction, AF episodes lasting more than 6 hours, a high ventricular rate during AF (>90 beats per minute for >6 hours in a day), OptiVol index (Medtronic Inc) >60 Ω, ventricular arrhythmias activating device therapies, or reduction of the percentage of biventricular pacing (<90%).

Information available in remote transmissions include, behind PA: arrhythmias, average heart rate, thoracic impedance, and daily heart rate variability. These data were compared among the four periods of the study.

We considered the variation of PA during the study period as the primary outcome. We also compared baseline characteristics and clinical events to data available by means of remote

transmissions. OptiVol episodes were considered as a surrogate of clinical events; the OptiVol algorithm was elaborated to identify thoracic fluid accumulation in an early phase and to monitor the duration of lower impedance episodes. Indeed, several studies have shown that variation in the content of fluid in the pulmonary vessels and tissues is associated with thoracic impedance changes [13,14]. Yu et al [15] demonstrated that during HF episodes, there is a relationship between pulmonary wedge pressure increase and the intrathoracic impedance.

### Statistical Analysis

Statistical analysis was performed using SPSS PASW Statistics 18 software. The Student *t* test or Mann-Whitney *U* test was used to compare continuous variables, whereas the Fisher exact test and Pearson *χ^2^* were used to compare categorical variables.

To assess independent predictors of PA modification, a multivariable logistic regression was performed. Statistical significance was set at the α=.05 level.

## Results

We enrolled 47 patients with HF; 6 (13%) patients were excluded because their daily activity was inferior to 0.5 hours/day before the lockdown period. Therefore, the study population included 41 patients with HF, 6 (15%) of whom were women. Ischemic etiology was present in 16 (39%) patients. In 17 (42%) patients, the device implanted was a CRT, whereas the remaining 24 (59%) patients had a single- or double-chamber ICD.

Every patient sent scheduled transmissions with data encompassing the whole study period; the overall number of transmissions was 176, including 61 (34.7%) alarm transmissions received during the study period.

**Figure 1:**
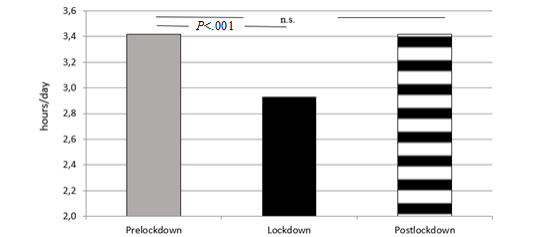
Daily physical activity amount in the three study periods. ns: not significant.

During the lockdown period, the mean variation in daily PA compared with that in the prelockdown period was –16.6% (*P*<.001). The mean daily PA decreased during the lockdown period (mean 3.4, SD 1.9 vs mean 2.9, SD 1.8 hours/day; *P*<.001) and then increased to a mean daily activity of 3.4 (SD 2.0) hours/day (*P*<.001 vs lockdown period). As shown in Figure 1, the average daily PA was not different before and after the lockdown period (–0.0007 hours/day; *P*=.99). Furthermore, PA postlockdown was not different to that in the same period of the previous year (mean 3.4, SD 2.0 vs mean 3.5, SD 2.0 hours/day; *P*=.40)

The baseline features of the population are summarized in Table 1.

No patient died during the study; two patients were admitted to hospital, both for noncardiovascular reasons and one of them for COVID-19.

The number of OptiVol episodes (OptiVol value >60 Ω) did not change significantly before, during, and after the lockdown period (9 vs 5 vs 5; *P*=.66). There was also no significant difference in the number of days with an OptiVol value >60 Ω among the three study periods (mean 5.7, SD 14.2 vs mean 5.1, SD 14.2 vs mean 3.3, SD 14.3 days, respectively; *P*=.44).

In the 3 months after the lockdown period, no significant difference was found in the number of nonsustained ventricular tachycardia (VT) episodes (20 vs 33 vs 42, respectively; *P*=.19). Similarly, no difference was found regarding the burden of atrial arrhythmias, when available: 8 days with at least 6 minutes of atrial arrhythmias in the period preceding the lockdown, 7 days during the lockdown, and 5 days in the period after lockdown (*P*=.12). Average heart rate also remained similar during the study (61 vs 60 vs 59 beats/minute, respectively; *P*=.97).

**Figure 2:**
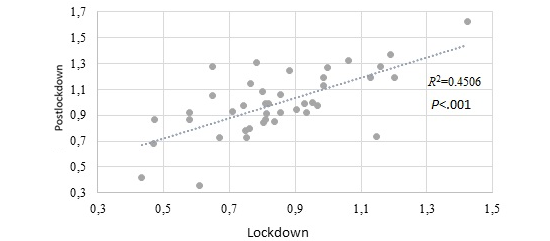
Correlation between relative variation in physical activity during the lockdown and postlockdown periods.

A significant correlation between the variation in daily exercise during the lockdown and the postlockdown period was found by means of linear regression analysis (*R^2^*=0.45; *P*<.001), as shown in Figure 2.

**Table 1:** Baseline characteristics of the study population and comparison of physical activity variation between groups.

Characteristic	All participants (N=41)	Physical activity variation during lockdown (%), mean (SD)			Correlation	*P* value
Gender (female), n (%)	6 (15)	–25 (28)		–12 (22)	—^a^	.37
Hypertension, n (%)	15 (37%)	–26 (24)		–6 (20)	—	.03
Diabetes mellitus, n (%)	7 (17)	–25 (9)		–14 (25)	—	.06
History of atrial fibrillation, n (%)	12 (29)	–18 (25)		–12 (23)	—	.52
Ischemic etiology, n (%)	16 (39)	–10 (20)		–18 (27)	—	.32
Type of device (CRT^b^), n (%)	17 (42)	–14 (20)		–17 (23)	—	.70
Serum creatinine (mg/dL), mean (SD)	1.12 (0.3)	—	—		0.06	.77
Hemoglobin (g/dL), mean (SD)	13.8 (1.8)	—	—		0.03	.13
LVEF^c^ (%), mean (SD)	39 (12)	—	—		0.02	.90
Age (years), mean (SD)	69.8 (12.6)	—	—		–0.06	.73

^a^Not applicable.

^b^CRT: cardiac resynchronization therapy.

^c^LVEF: left ventricular ejection fraction.

The study population was then separated in two groups: group A included all patients that showed daily activity variation above the average value (–0.7%) after the lockdown compared to the baseline and group B included those with a variation lower than the average.

As shown in Table 2, the patients in group B (24/41, 59%) had a higher prevalence of hypertension, history of atrial fibrillation, and a lower hemoglobin level compared with those of patients in group A. In addition, the reduction of PA during the lockdown period was significantly greater in group B than that in group A patients (mean –24.4%, SD 17.1% vs mean –3.2%, SD 21.0%; *P*=.001).

Patients in group B had a higher number of alarm transmissions compared with that of patients in group A (mean 3.4, SD 1.0 vs mean 2.5, SD 0.6; *P*=.05) due to VT, atrial arrhythmias, or OptiVol episodes; no specific alarm trigger defined a clear difference in the two main groups.

The only significant predictor of PA variation in the postlockdown period was the lockdown to prelockdown PA ratio (odds ratio 2.26, 95% CI 1.0-5.22; *P*=.05).

**Table 2:** Comparison between patients with reduced and fully recovered physical activity after the lockdown.

Characteristic	Physical activity recovered (n=17)	Physical activity reduced (n=24)	*P* value
Age (years), mean (SD)	65.7 (14.4)	73.7 (14.5)	.08
Gender (female), n (%)	1 (6)	5 (201)	.18
Hypertension, n (%)	3 (18)	12 (50)	.03
Diabetes mellitus, n (%)	1 (6)	6 (33)	.08
History of atrial fibrillation, n (%)	2 (12)	10 (42)	.04
Ischemic etiology, n (%)	6 (35)	10 (42)	.68
LVEF^a^ (%), mean (SD)	37 (15)	42 (7)	.26
Serum creatinine (mg/dL), mean (SD)	1.19 (0.3)	1.04 (0.3)	.20
Hemoglobin (g/dL), mean (SD)	14.7 (1.5)	12.9 (1.7)	.01
Type of device (CRT^b^), n (%)	10 (59)	7 (29)	.06

^a^LVEF: left ventricular ejection fraction.

^b^CRT: cardiac resynchronization therapy.

## Discussion

This study investigated the effect of the COVID-19 lockdown on PA in patients with HF, focusing both on the lockdown period and on the postlockdown period. Similar studies had limited the comparison between prelockdown and lockdown periods [7] or included unselected patients [8], whereas information on postlockdown changes in patients with HF are lacking.

The main findings of this study are that the mean level of exercise in the period before the lockdown was recovered after the lockdown period, but the reduction in PA in the postlockdown period compared to the prelockdown period is a function of the entity of the PA reduction during the lockdown period.

These findings are partially in agreement with those reported by Cunha et al [11], showing difficult PA recovery in patients with low basal PA; however, they used a shorter study period and did not consider PA reduction during lockdown as a possible predictor of PA recovery failure.

As expected, in our study, the average daily PA decreased during the lockdown period, in line with previous reports [9,10]. PA then recovered, increasing to the same level as found in the prelockdown period. In a similar study, Bertagnin et al [16] performed a week-to-week analysis including 211 patients, and found that PA decreased (–25.9%, *P*<.001) during the prelockdown period. Of note, patients’ perceptions about PA showed a very low correlation with remote monitoring–assessed PA levels (*R^2^*=0.035, *P*=.04) [16].

Reduction of exercise and delay in medical visits during the holidays were suggested as some of the possible causes of the higher incidence of increased HF admission after the holidays [17]. According to this observation, in our study, the postlockdown period was also compared with the same period of the previous year. The mean PA was similar and no difference in hospitalization or death was found; however, the study is underpowered to state any definitive conclusion on such clinical outcomes.

The relationship between the level of PA and HF outcomes is controversial. Patel et al [18] showed that higher PA levels were associated with a lower frequency of HF episodes. Moreover, a reduction of daily PA has been associated with HF worsening and higher mortality in other studies [19,20]. Overall, exercise was shown to be a possible predictor of HF morbidity and mortality. In this study, patients with stable PA and those with reduced average daily activity did not show any significant differences in the number and duration of OptiVol episodes. However, the larger the reduction of PA during the lockdown period, the higher the number of alarm episodes, suggesting a negative effect of PA reduction, independent of the activation of a specific alarm. Unfortunately, this study was underpowered to investigate this issue.

Factors such as hypertension, anemia, and diabetes were significantly associated with a worse exercise recovery after the lockdown period. This could suggest that patients with more risk factors should be strictly monitored to endorse their PA recovery, because PA is an important step in lifestyle change for these patients.

We acknowledge that the study enrolled a very selected group of patients to carefully investigate a specific feature (ie, PA) in the three considered periods. Thus, results requiring larger samples to be demonstrated could not be found, as in the case of ventricular arrhythmias whose highest incidence was a mean of 0.09 (SD 1.2) episodes per patient per week in a previous study on the COVID-19 pandemic and remote monitoring [21].

All patients enrolled in this study were followed by means of a specific software made by a single manufacturer. Other devices have the capability to estimate PA; however, we focused on a single tool of remote monitoring to minimize diagnostic differences between different software types.

Both CRT and ICD patients were enrolled. These two groups of patients could have different perspectives, because a larger clinical benefit should be expected in CRT patients by the device itself [22]. Nevertheless, in the setting of this study that evaluated mainly PA in a limited period, the CRT per se should not be expected to have a significant role in that regard; the daily exercise level was indeed unchanged in the postlockdown period compared with that for the same period in the previous year.

In addition, the effect of COVID-19 infection was not assessed due to clinical and diagnostic limitations. The former depends on the low number of patients included in the study and, in particular, of patients with symptoms due to ascertained COVID infection; nevertheless, the low number of patients infected and the absence of death due to COVID-19 is a positive result in this high-risk population. The latter depends on the diagnostic strategy implemented in Italy during the first months of the pandemic that excluded asymptomatic patients from COVID-19 diagnostic research. Thus, the population could include patients who were infected, although the role of infection in the modification of exercise attitude and tolerance could not be evaluated.

In conclusion, an excessive reduction of PA due to lockdown measures in patients with HF decreased the tolerance to exercise, whose consequences should be investigated in larger studies. Patients with a higher prevalence of comorbidities appear to be at higher risk to fail in achieving full recovery of the basal PA level.

Implementation of a remote monitoring strategy could help patients with HF maintain an adequate level of PA in a critical period.

## Abbreviations

AF: atrial fibrillation
CRT: cardiac resynchronization therapy
HF: heart failure
ICD: implantable cardioverter defibrillator
PA: physical activity
VT: ventricular tachycardia
